# The Protective Effect of Yi Shen Juan Bi Pill in Arthritic Rats with Castration-Induced Kidney Deficiency

**DOI:** 10.1155/2012/102641

**Published:** 2012-04-05

**Authors:** Hongyan Zhao, Jian Li, Xiaojuan He, Cheng Lu, Cheng Xiao, Xuyan Niu, Ning Zhao, Dahong Ju, Aiping Lu

**Affiliations:** ^1^Institute of Basic Theory of Chinese Medicine, China Academy of Chinese Medical Sciences, Beijing 100700, China; ^2^Preclinical College, Beijing University of Chinese Medicine, Beijing 100029, China; ^3^Institute of Basic Research in Clinical Medicine, China Academy of Chinese Medical Science, Beijing 100700, China; ^4^China-Japan Friendship Hospital, Beijing 100029, China

## Abstract

Androgens have been linked to the onset, severity, and progression of rheumatoid arthritis (RA). In traditional Chinese medicine (TCM), the most common pattern in RA is kidney deficiency, which partly corresponds to a low sex hormone state. In this study, TCM kidney deficiency was induced in male Sprague-Dawley rats with castration surgery, and a TCM preparation, Yi Shen Juan Bi Pill (YJB), was used to treat collagen induced arthritis (CIA) rats with castration. Metabolomic technique was used to evaluate the pharmacological mechanism in castrated CIA rats treated by YJB. The results showed that castration significantly increased the severity of the arthritis in rats but was ameliorated by YJB. Its pharmacological mechanism was partially associated with lipid metabolites involving free fatty acid (FFA) and lysophosphatidylcholine (LPC). *In conclusion*, the experimental results demonstrate the protective effect of YJB on the TCM kidney deficiency pattern induced by androgen deficiency in CIA rats and support that YJB should be used for the clinical treatment of RA with TCM kidney deficiency pattern.

## 1. Introduction

Rheumatoid arthritis (RA) is a systemic autoimmune disease that primarily presents as chronic symmetric polyarthritis associated with inflammation and cartilage destruction. Epidemiological data suggest that approximately 1% of the world's population is afflicted with RA [1]. It is hypothesized that hormonal factors play a pathogenic role in RA onset [2–4]. 

In traditional Chinese medicine (TCM), RA is categorized as *Bi Zheng* (Bi syndrome or blockage syndrome). The TCM kidney deficiency pattern is the most common pattern addressed to manage RA; thereby reinforcing the TCM kidney is an important therapeutic target for RA [5, 6]. The Chinese patent drug Yi Shen Juan Bi pill (YJB) was approved (no. Z10890004) and has been marketed in pill form in China since 1987. The formula was prepared by the National TCM master professor Zhu Liangchun, and YJB has been proven as an effective treatment for RA with TCM kidney deficiency pattern [7]. YJB has been shown to ameliorate RA symptoms and to decrease the erythrocyte sedimentation rate (ESR), as well as C-reactive protein (CRP) and rheumatoid factor (RF) levels [8]. Recent studies have elucidated the mechanisms used by YJB; specifically, it significantly decreased prostaglandin E (PGE) and upregulated the pro-apoptotic family member Bax in rat synovium and decreased the production of peritoneal macrophage-derived tumor necrosis factor-alpha, interleukin 1 and nitric oxide [9–11]. Interestingly, a clinical trial suggested that YJB combined with methotrexate was effective in treating elderly onset RA, which was characterized by low plasma testosterone [12]. However, the pharmacological activity and mechanisms of YJB in the treatment of RA with kidney deficiency pattern are not clear. 

TCM kidney deficiency was reported to be induced in castrated rats [13]. It has also been hypothesized that low levels of sex hormones partially correspond to TCM kidney deficiency pattern [14]. Therefore, castrated rats are a suitable animal model for TCM kidney deficiency [13, 15]. 

The formulation of the YJB consists of complex components; therefore, it is challenging to understand therapeutic mechanisms with conventional methods. Recently metabolomic approaches have been utilized to understand pharmacological mechanisms of related compound Chinese herbs [16, 17]. In the present study, we established collagen-induced arthritis (CIA) in rats to evaluate the effect of YJB treatment on inflammatory responses in normal CIA rats and CIA rats with castration-induced TCM kidney deficiency pattern. We also obtained metabolic profiles of plasma from CIA rats with TCM kidney deficiency with or without YJB treatment to supply further evidence for the clinical application of YJP in the treatment of RA with TCM kidney deficiency.

## 2. Materials and Methods

### 2.1. Materials

YJB was obtained from GMP-approved Jiangsu Zhengda Qingjiang Pharmaceutical Co., Ltd. HPLC-grade acetonitrile and formic acid was purchased from Merck (USA). Freund's incomplete adjuvant and bovine type II collagen were purchased from Sigma-Aldrich (MO, USA). IL-6 (BMS625) and IL-10 (BMS629) assay kits were purchased from Bender (USA). Ultrapure water was from a Milli-Q50 SP Reagent Water System (Millipore Corporation, USA).

### 2.2. Animal Handling Procedure

Male Sprague-Dawley (SD) rats (150 ± 10 g) were purchased from the Institute of Experimental Animals in the Chinese Academy of Medical Science (rodent license no. SYXK 11-00-0039). The rats were housed under standard laboratory conditions, and food and tap water were provided ad libitum. Experimental procedures were reviewed and approved by the Animal Care and Use Committee in the China Academy of Chinese Medical Sciences before the animal experiments were carried out. 

Castration was performed according to standard surgical procedures under pentobarbital anesthesia. In brief, a single incision was made in the scrotal skin, and the testicles were squeezed out with gentle pressure. The spermatic cord was ligated with chromic catgut, and scrotal incisions were treated as open wounds.

Arthritis was induced as previously described, 4 weeks after castration [18, 19]. Briefly, rats were intradermally injected at the base of tail with 100 *μ*g of bovine type II collagen in 0.05 M acetic acid emulsified with an equal amount of incomplete Freund's adjuvant. The rats were given a booster with the same preparation 7 days after the primary immunization. 

### 2.3. Experimental Groups and Drug Treatment

The experimental groups (*n* = 10) were as follows: (1) normal control (NC), (2) collagen-induced arthritis (CIA), (3) castration-induced kidney deficiency arthritis, and (4) castrated CIA rats with YJB treatment. On day 15 after the primary immunization, one group began receiving daily YJB (2.4 g/kg body weight) by intragastric administration for 14 days. The dose was based on the clinical application dosage of 24 g per day per 60 kg body weight. Other groups were treated with an equal volume of distilled water as a vehicle control.

### 2.4. Arthritis Assessment

CIA rats were assessed for disease severity every 2 days after the booster immunization. Arthritis severity was expressed as mean arthritic index on a 0 to 4 scale according to the conventional method [20]. In addition, joint tissue histopathology was assessed with H & E staining. Inflammation, pannus, cartilage damage, and bone damage were scored on scales from 0 to 3 (0: absent; 1: weak; 2: moderate; 3: severe) [21].

### 2.5. Measurement of IL-6 and IL-10

Serum levels of IL-6 and IL-10 were measured with ELISA according to the manufacturer's instructions. Briefly, blood serum was harvested after the rats were sacrificed and diluted 1 : 10. The absorbance was read at 450 nm using a microplate reader. Samples and standards were analyzed in triplicate.

### 2.6. Anti-Col II Antibody Measurement

The serum level of anti-Col II antibody was measured by ELISA. Briefly, collagen was dissolved to a final concentration of 10 *μ*g/mL in acetic acid (0.1 moL/L), and the resulting solution was applied to 96-well flat-bottomed microtiter plates at 4°C overnight. Next, the wells were incubated with 0.5% ovalbumin at room temperature for 1 h to reduce nonspecific binding. After washing with phosphate-buffered saline containing Tween, diluted test serum and standards were added to the Col II-coated wells. Next, the biotin-conjugated goat affinity-purified antibody to rat IgG and sequentially streptavidin-HRP were added. The reaction was terminated by the addition of stop buffer, and absorbance was measured at 450 nm.

### 2.7. HPLC-Q-TOF-MS Conditions

HPLC-Q-TOF-MS analysis was performed on a Water-Q-TOF Micro MS system coupled with an electrospray ionization (ESI) source (Water Technologies, UK). Samples were separated on an Eclipse plus C18 column with the column temperature set at 35°C. Data were collected in full scan mode from 100 to 1000 m/z from 0 to 30 min. The standard sample ran six times continuously to confirm the stability of the method. The mass detection was operated in both positive and negative ion modes (flow rate: 8 L/min, gas temperature: 250°C, pressure of nebulizer gas: 35 psig, Vcap: 3 kV, fragmentor: 160 V, skimmer: 65 V). Target MS analysis was used to identify potential biomarkers.

### 2.8. Metabolomic Data Analysis

The raw data were analyzed with MarkerLynx software (Waters, UK) for peak deconvolution and alignment. The parameters were as follows: mass tolerance was set at 0.05 Da, peak width was set at ≥1.5%, baseline noise elimination was set at level 4, and the mass window was set at 0.1 min. The data were combined into a single matrix by aligning peaks with the same mass/retention time (0.3–12 min) from each data file in the dataset, along with their associated normalized intensities. Principal component analysis (PCA) was performed with SIMCA-P software (Version 12.0, UMETRICS AB, Box  7960, SE 90719, Umea, Sweden) to visualize general clustering for further identification of differentially expressed metabolites that might account for the separation between YJB-treated rats and other groups.

### 2.9. Statistical Analysis

All of the quantitative data analyses were performed using SPSS 11.5 software package for Windows. Significance was determined with one-way analyses of variance (ANOVAs) followed by Student's *t*-tests. Results were expressed mean ± SD. *P* values less than 0.05 were considered significant.

## 3. Results

### 3.1. Change on Sex Hormone and Arthritic Evaluation

**Figure 1:**
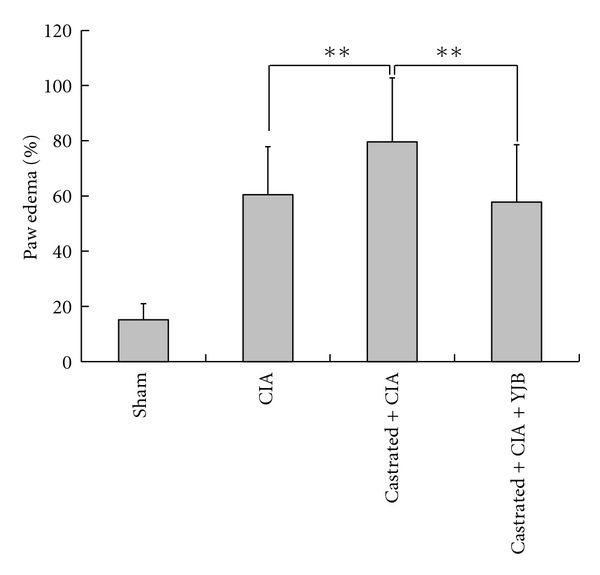
Paw edema by treatment (*n* = 10/group). YJB treatment suppresses paw swelling in castrated arthritic rats. Data are expressed as means ± SD (**P < 0.01).

**Figure 2:**
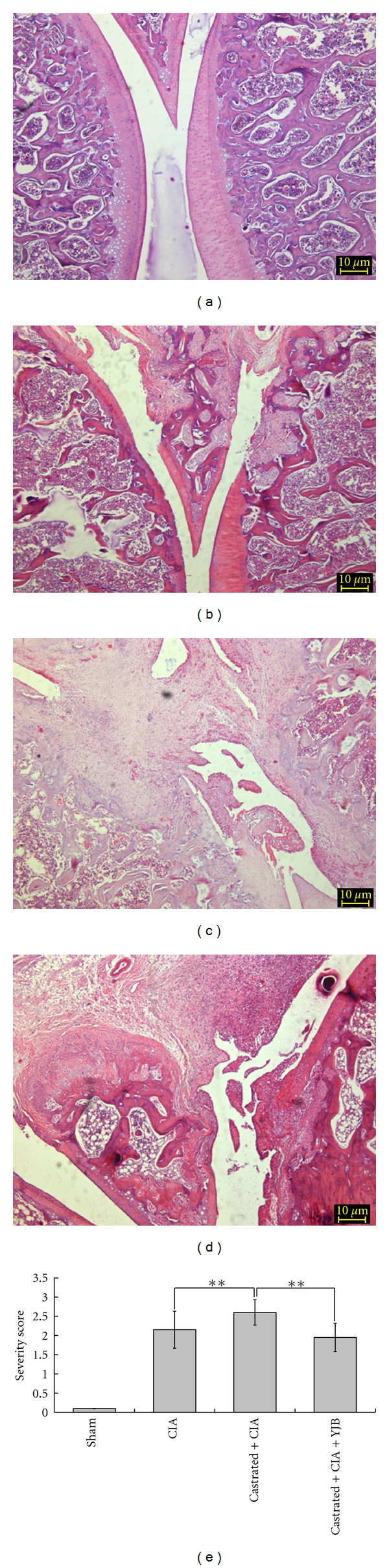
Histological evaluation of the joint tissue. Paraffin sections of knee joints were stained with H & E. Scale = 100 *μ*m. (a) Sham group treated with distilled water; (b) CIA rats treated with distilled water; (c) castrated arthritic rats treated with distilled water; (d) castrated arthritic rats treated with YJB; (e) pathological severity score. Pathological changes were scored on a 1 to 3 scale. Data are expressed as means ± SD (**P < 0.01).

Castration-induced TCM kidney deficiency can significantly reduce dihydrotestosterone, testosterone, and estradiol serum levels in rats. No significant differences in these hormone levels were detected in the YJB-treated group compared to castrated arthritic control group (Supplementary Data S1 available online at doi:10.1155/2012/102641). Arthritis was reproducibly induced in both normal rats and castration-induced kidney deficiency rats that were given collagen combined with ICFA (data not show). The results showed significantly increased paw edema in castrated arthritic rats, which was suppressed by YJB administration (Figure 1). Histological evaluation of joint tissue demonstrated that, compared with CIA rats, castrated CIA rats showed extensive cartilage erosion, fibroplasia, and synovial membrane thickening (Figure 2(c)). Clinical evaluations and histological studies demonstrated that the severity score was higher in castrated CIA rats compared to normal CIA rats (*P* < 0.01, Figure 2(e)); YJB treatment reduced degradation and resulted in a significantly lower severity score compared to castrated CIA rats (*P* < 0.01, Figures 2(d) and 2(e)).

### 3.2. Serum Levels of Anti-Collagen Type II, IL-6, and IL-10

**Figure 3:**
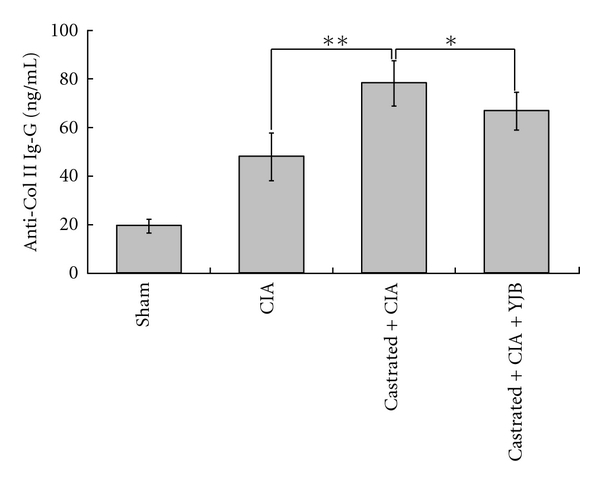
Changes of anti-Col II IgG in serum (*n* = 10). The level of anti-Col II was measured by ELISA. Data are expressed as means ± SD (**P < 0.01, *P < 0.05).

**Figure 4:**
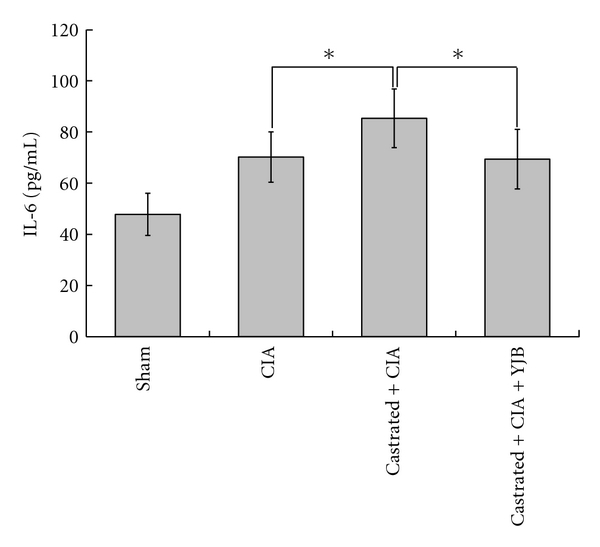
Changes of IL-6 in serum (*n* = 10). The level of IL-6 was measured by ELISA. Data are expressed as means ± SD (*P < 0.05).

**Figure 5:**
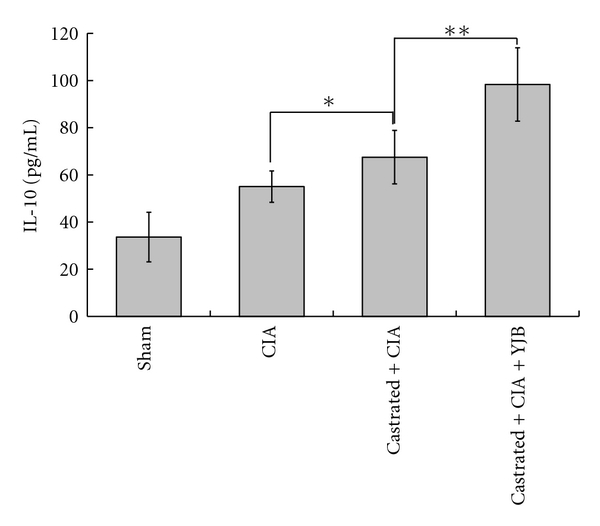
Changes of IL-10 in serum (*n* = 10). The level of IL-10 was measured by ELISA. Data are expressed as means ± SD (**P < 0.01, *P < 0.05).

Castrated CIA rats developed a significantly higher immune response in terms of antibody generation against type II collagen compared to the CIA control (*P* < 0.01). The antibody titers of castrated CIA rats were significantly attenuated by YJB administration (*P* < 0.05, Figure 3). Arthritis induction caused increased serum levels of IL-6. While castrated CIA rats exhibited an on-going IL-6 increase (*P* < 0.05), this increase was significantly suppressed by YJB treatment (*P* < 0.05, Figure 4). However, the opposite was true for IL-10. Castrated CIA rats treated with YJB had significantly higher levels of IL-10 level compared to castrated CIA rats (*P* < 0.01, Figure 5).

### 3.3. Metabolite Identification

**Figure 6:**
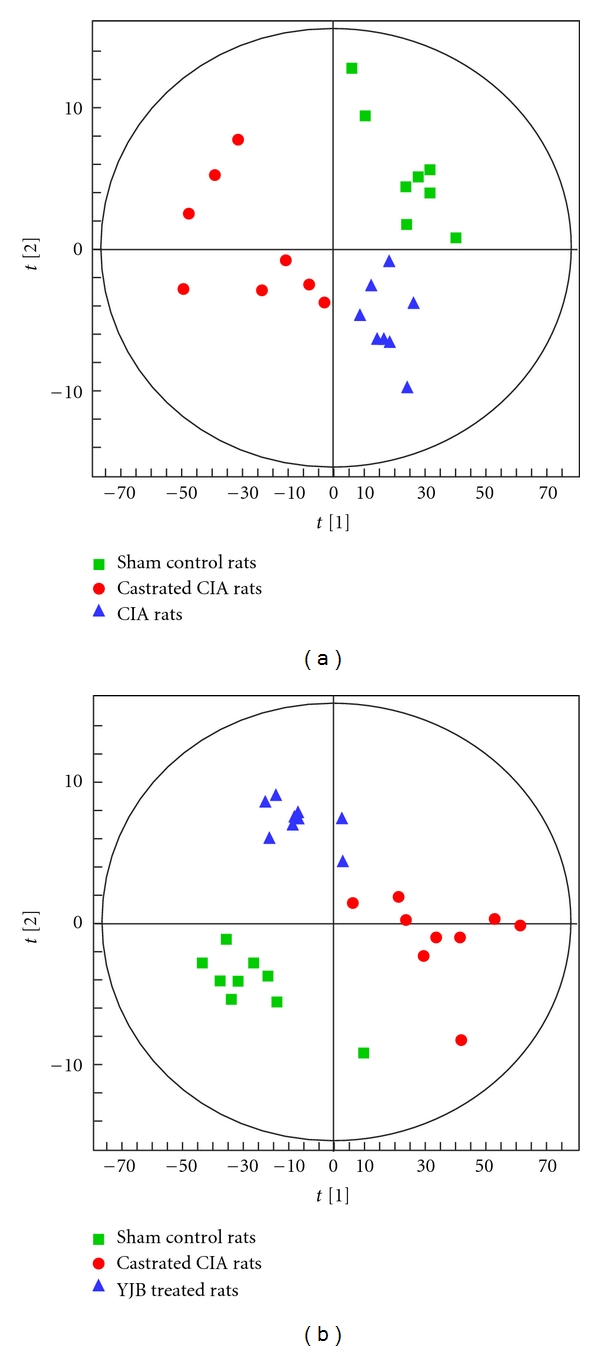
(a) Principal component analysis (PCA) scores (component 1 versus component 2) of serum metabolites derived from sham rats (■), CIA rats (▲), and castrated CIA rats (●). The 3 groups were clearly separated. (b) Principal component analysis (PCA) scores (component 1 versus component 2) of serum metabolites derived from sham rats (■), castrated CIA rats (●), and castrated CIA rats treated with YJB (▲). A clear separation of the score spot was observed in the three groups.

In order to understand the role of the castration-induced kidney deficiency in arthritis onset, we performed serum metabolic profiling of normal control rats, CIA control rats, and castrated arthritic rats. Figure 6(a) shows a clear separation trend of metabolites between normal rats, CIA rats, and castrated CIA rats using unsupervised analysis of PCA. This result suggests that castration-induced kidney deficiency perturbed the metabolic profile of castrated arthritic rats.

To ascertain the effect of YJB on serum metabolite perturbation in castrated arthritic rats, we examined the metabolic profiles of serum in normal rats, castrated CIA rats, and castrated CIA rats treated with YJB. Figure 6(b) shows a clear separation of scores between normal control rats and castrated arthritic rats, and a clear separating trend between castrated CIA rats and castrated CIA rats treated with YJB. This finding suggests that YJB could ameliorate the pathological state induced by arthritis and castration. These results support the hypothesis that YJB has a therapeutic effect on arthritic rats with castration-induced TCM kidney deficiency pattern.

**Figure 7:**
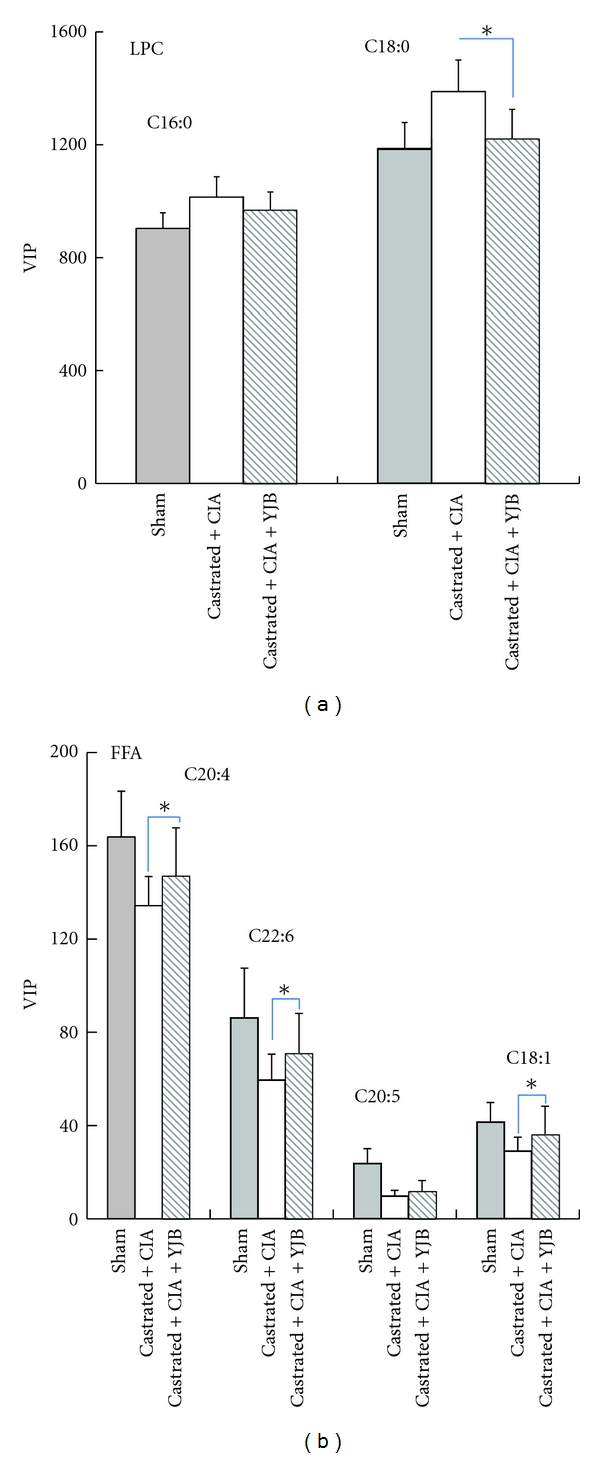
The value of variable important parameters (VIPs) for (a) LPC and (b) FFA by groups *P < 0.05 versus the castrated CIA group (one-way ANOVA, followed by Student's *t*-test).

Over 300 peaks were obtained using LC-TOF-MS analytical protocols coupled with a software-based peak deconvolution procedure. Student's *t*-tests were performed on all metabolites. The variables selected were those with statistically significant differences (*P* < 0.05) between normal control rats, castrated CIA rats, and YJB-treated rats. A total of 20 individual metabolites were significantly different. Compound identification was performed with commercially available authentication standards. Among these perturbed variables, 14 (7 upregulated and 7 downregulated) were predicted by comparing the accurate MS and MS-MS fragments with metabolites found in databases (http://metlin.scripps.edu/; http://www.hmdb.ca/) that were later confirmed with commercial standards. Most of the metabolites were lipids, such as LPC and FFA (Supplementary Data S2). The statistical results demonstrated that YJB may downregulate LPC (Figure 7(a)) and upregulate FFA in the serum of castrated CIA rats (Figure 7(b)). 

## 4. Discussion

A major finding of this study is that CIA rats with castration-induced TCM kidney deficiency can develop severe arthritis, and YJB could have a therapeutic effect in castrated CIA rats. This result supports the clinical application of YJB in the treatment of RA patients with TCM kidney deficiency pattern. 

It is well known that sex hormones, which are important factors in TCM kidney deficiency [5–7], might have a pathogenic role in RA onset. It has been reported that RA occurs 3-4 times more frequently in women than in men [2]. Furthermore, men with RA have lower serum testosterone levels than healthy men [3], and male gender has been found to be a major predictor of remission in early RA [4]. In our results, castrated male rats developed severe arthritis after collagen immunization. 

YJB is a TCM compound that is hypothesized to reinforce kidney function. YJB consists of 20 medicinal materials, including *Fructus Xanthii, Herba Cistanchis, Radix angelica, Radix Rehmanniae, Pheretima, Radix Glycyrrhizae, Rhizoma Drynariae, Polygoni Cuspidati, Caulis Spatholobi, Bombyx Batryticatus, Herba Erodii, Herba Pyrolae, Allomyrina dichotoma, Scorpio, Radix Rehmanniae Preparata, Eupolyphaga Seu Steleophaga, Scolopendra, Zaocys (stir-fried with wine), Cynanchi Paniculati, Herba Aristolochiae, Rhizoma Corydalis, Herba Epimedii, Nidus Vespae (stir-baking), and Nidus Vespae*. One study reported that YJB effectively treated arthritis in rats [11], and our results showing that YJB affects the balance of proinflammatory cytokines IL-6/IL-10 support its anti-CIA activity, further. IL-10 and IL-6 are important in the development of RA [22]. The exogenous addition of IL-10 in vivo has been shown to affect the immunopathological processes involved in RA, although the outcome of clinical studies using IL-10 was disappointing [23]. IL-6 is closely associated with the pathological process of RA. The evidence suggests that, in contrast to IL-6, IL-10 plays an active role in ameliorating arthritis caused by degeneration. In our study, YJB treatment increased IL-10 and decreased IL-6 in the serum of arthritic rats with castration-induced TCM kidney deficiency. 

Another major finding in this study was the identification of 2 kinds of metabolites, including LPC and FFA, which are directly relevant to lipid metabolism. According to previously published data and biochemical databases (e.g., KEGG and METLIN), we demonstrated that FFA and LPC are critical intermediates of fatty acid metabolism [24]. As shown in Figure 7, decreased LPC levels were observed in the serum of YJB-treated rats. Researchers have demonstrated that LPC is involved in inflammatory disease pathogenesis, and LPC levels could increase in response to reactive oxygen species (ROS) and inflammatory conditions, such as RA, lung infection, diabetes, and liver injury [25–27]. The decreased level of LPC in response to YJB treatment could disturb choline and cholesterol metabolism, which might be the pharmacological mechanism of the compound. Along with decreased LPC, the increased level of FFA suggests increased acetyl-CoA, an important substrate in the TCA cycle, which is critical for energy production. The metabolic profile implies that YJB may influence lipid metabolism regulation in CIA rats with castration-induced TCM kidney deficiency. 

Androgens are involved in the pathogenesis of RA to a surprising degree. Their modulation of the activity of cells involved in the immune inflammatory response is dependent on the androgen/estrogen ratio and concentration [28]. Clinical studies have revealed the effects of androgens on the treatment of autoimmune and chronic inflammatory diseases, such as RA, SLE, and tumors [29]. Unfortunately, conclusions from studies of sex hormone therapy were often paradoxical [30, 31]. The biological significance of the association between androgens and RA remains unclear. In this study, we limit the conclusion to the effect of YJB, which protected against castration-induced androgen deficiency in arthritic male rats by downregulating IL-6 (a proinflammatory cytokine), upregulating IL-10 (an anti-inflammatory cytokine), and regulating lipid metabolism. Honestly, the major limitation of this study is that the metabolomic data were not fully collected and analyzed. Because lipid metabolism is a likely mechanism of YJB in the treatment of CIA rats, a more thorough metabolomic study is necessary.

## 5. Conclusion

In conclusion, castration-induced TCM kidney deficiency significantly increased the severity of arthritis in rats. YJB had protective effects on CIA rats with castration-induced TCM kidney deficiency, and its pharmacological mechanism likely involved lipid metabolites, including FFA and LPC. These results suggest that YJP should be used for the treatment of RA with TCM kidney deficiency pattern. 

## Supplementary Material

Castration induced TCM kidney deficiency can significantly reduce the level of dihydrotestosterone, testosterone and estradiol in serum of rats. No significant difference on the levels of hormones was detected in YJB treated group compared to castrated arthritic control group.
