# Supplementary material for: The Protective Effect of Yi Shen Juan Bi Pill in Arthritic Rats with Castration-Induced Kidney Deficiency
Source: Evid Based Complement Alternat Med. 2012 Apr 5;2012:102641. doi: 10.1155/2012/102641 (PMC3329149; doi:10.1155/2012/102641)
Supplement: Supplementary file 1 — Castration induced TCM kidney deficiency can significantly reduce the level of dihydrotestosterone, testosterone and estradiol in serum of rats. No significant difference on the levels of hormones was detected in YJB treated group compared to castrated arthritic control group. [file 102641.f1.pdf]

S 1. Hormone levels in serum from male rats by group (mean±SD)

Group	Dihydrotestosterone (pg/ml)	Testosterone (ng/ml)	Estradiol (pg/ml)
Sham control rats	162.49±24.25	0.86±0.07	44.16±9.38
CIA rats	147.47±28.90	0.74±0.10*	39.42±9.98
CIA rats with castration	76.07±5.34**	0.39±0.36**	14.15±4.88**
Castrated CIA with YJB treatment	72.01±3.57**	0.40±0.20**	15.13±4.25**

* ***p***<0.05, ** ***p***<0.01 ***vs.*** sham control group
