# Supplementary material for: The Protective Effect of Yi Shen Juan Bi Pill in Arthritic Rats with Castration-Induced Kidney Deficiency
Source: Evid Based Complement Alternat Med. 2012 Apr 5;2012:102641. doi: 10.1155/2012/102641 (PMC3329149; doi:10.1155/2012/102641)
Supplement: Supplementary file 2 [file 102641.f2.pdf]

S 2. Significantly changed metabolites in the male rat's serum when disturbed by YJB

t	*m/z*	*Ionization mode*	*Metabolite*	*Peak area (mean±SD)*				***P*-value**	
				Sham control	Castrated CIA	YJB treated		Sham control *vs.* Castrated CIA	YJB-treated *vs.* Castrated CIA
15.4	496.3	ESI+	LPC C16:0	903.7±55.6	1014.5±72.2	967.3±65.4	↓	0.003	0.17
16.0	522.3	ESI+	LPC C18:1	222.3±21.8	179.1±17.8	194.7±30.0	↑	0.000043	0.2
17.0	525.3	ESI+	Isotope of LPC C18:0	312.9±28.6	363.7±51.0	303.5±34.4		0.03	0.01
17.1	524.3	ESI+	LPC C18:0	1185.4±93.5	1388.7±111.4	1219.9±105.7	↓	0.001	0.005
19.2	303.2	ESI-	FFA C20:4	163.8±19.6	134.3±12.5	146.9±20.8	↑	0.002	0.14
15.4	566.3	ESI-	LPC C18:1 $[M^{+}HCOO]^{-}$	251.4±25.8	218.5±12.9	228.6±15.1	↑	0.006	0.18
15.4	506.3	ESI-	LPC C18:1 $[M^{-}CH_3]^{-}$	281.4±33.5	235.8±29.6	238.4±20.4		0.009	0.83
19.0	327.2	ESI-	FFA C22:6	86.1±21.4	59.5±11.1	71.0±17.1	↑	0.005	0.11
14.5	528.3	ESI-	LPC C20:4 $[M^{-}CH_3]^{-}$	250.6±24.3	222.9±22.7	197.7±10.6	↓	0.03	0.008
14.7	540.3	ESI-	LPC C16:0 $[M^{+}HCOO]^{-}$	555.5±60.9	688.5±79.2	622.3±29.3	↓	0.002	0.04
16.5	569.3	ESI-	Isotope of LPC C18:0 $[M^{+}HCOO]^{-}$	160.8±8.6	235.9±40.0	192.1±23.4		0.000041	0.014
16.5	568.3	ESI-	LPC C18:0 $[M^{+}HCOO]^{-}$	585.8±35.1	786.8±124.5	664.0±41.2	↓	0.001	0.02
16.5	508.3	ESI-	LPC C18:0 $[M^{-}CH_3]^{-}$	507.1±33.6	639.7±79.2	542.6±51.4	↓	0.000078	0.007
16.4	509.3	ESI-	Isotope of LPC C18:0 $[M^{-}CH_3]^{-}$	131.9±9.6	189±36.0	148.5±6.8		0.001	0.097
14.7	480.3	ESI-	LPC C16:0 $[M^{-}CH_3]^{-}$	600.5±65.0	727.3±75.7	666.5±30.4		0.002	0.048
14.4	241.4	ESI-	unknown	49.7±19.0	15±5.3	11.0±2.1		0.001	0.06
14.5	588.3	ESI-	LPC C20:4 $[M^{+}HCOO]^{-}$	241.8±21.9	208.3±21.9	186.2±12.9	↓	0.007	0.02
13.8	478.3	ESI-	LPC C16:1 $[M^{-}CH_3]^{-}$	58.2±10.7	41±6.2	51.7±7.5	↑	0.000092	0.005
18.1	301.2	ESI-	FFA C20:5	23.6±6.5	9.7±2.5	11.9±4.5	↑	0.0000021	0.22
20.6	281.2	ESI-	FFA C18:1	41.5±8.4	29.0±6.0	36.2±12.1	↑	0.003	0.13
